# Raqeeb, Haastrup and Evans: Seeking Consistency Through a Distributive Justice-Based Approach to Limitation of Treatment in the Context of Dispute

**DOI:** 10.1017/jme.2022.21

**Published:** 2022-01-01

## 1 Introduction

A number of recent high profile court cases in the United Kingdom involving mechanical ventilation of critically ill children highlight the challenges of asking courts to identify a child’s best interests. Courts sometimes struggle to identify the child’s ‘best interests.’ As a result, they may reach inconsistent conclusions. There have been recent proposals to change the law applying to disputes about treatment.^1^ We argue that such changes are not necessarily required, but defend a different approach to the ethical basis for decision-making and the role of the court.

We will draw on the recent case of *Tafida Raqeeb v Barts NHS Foundation Trust*. We argue that the apparent inconsistency between this decision and other recent cases highlights the subjectivity and formidable ethical challenge of determining the best interests of a minimally conscious young child. Moreover, we argue that the decision in this case ignored a central and relevant ethical consideration: resource allocation and distributive justice. Decisions in the NHS must be made to ensure limited medical resources are allocated ethically, efficiently and effectively.^2^ This must inform decision making.

We argue that rather than turning to the courts to identify what is in a child’s best interests, hospitals should make decisions about when it is appropriate to provide mechanical ventilation under conditions of distributive justice. If a hospital determines mechanical ventilation would not be an appropriate use of resources, parents may seek judicial review of whether this decision is lawful or they may privately fund treatment elsewhere. If the hospital is concerned that treatment arranged by the parents would cause significant harm to the child, they should apply to the court for a care order or a supervision order. We contend that this approach better reflects the role and expertise of the relevant parties. It would also prevent the courts from relying on an outdated concept that a judge (acting in the role previously fulfilled by the monarch as the wise parent) is well placed to objectively identify the best interests of a child and indeed whether it is in the child’s best interests to die.^3^

## 2 Tafida Raqeeb

The recent case of *Raqeeb* involved a five year old girl, Tafida Raqeeb, who suffered bleeding on her brain that resulted in extensive and irreversible brain damage.^4^ After six months, Tafida remained in a minimally conscious but medically stable state. Tafida’s doctors concluded that although she could be kept alive on mechanical ventilation, this would be of no benefit to her and treatment should be withdrawn. Tafida’s parents did not accept this opinion. They found doctors in Italy who were willing to continue to provide mechanical ventilation and who were also prepared to undertake a tracheostomy to allow Tafida to receive home ventilation. They believed that there was a possibility of some neurological improvement over the next year, but could not say whether this would be better for Tafida.^5^

When Tafida’s parents requested that Tafida be transferred to Italy for further treatment, the NHS Trust refused. Tafida’s parents made an application seeking judicial review of the Trust’s decision not to agree to the transfer of Tafida. The Trust, meanwhile, sought a declaration that it would be lawful to withdraw life sustaining treatment from Tafida. Justice MacDonald held that the Trust’s decision to refuse transfer was unlawful, because they failed to consider Tafida’s right to free movement in the European Union. However, the judge acknowledged that if the Trust had considered Tafida’s Article 56 rights, it would have reached the same conclusion (that the request should be declined pending review by the court).^6^ Of greater consequence was Justice MacDonald’s decision that ongoing ventilation was in Tafida’s best interests and that she must be provided with mechanical ventilation.^7^ What was acknowledged, but not addressed, was that once long term mechanical ventilation was established in Italy, Tafida’s parents could choose to transfer her back to England and at that point English doctors may be placed in a position of continuing treatment that they do not believe is in Tafida’s best interests.

Justice MacDonald came to the conclusion that mechanical ventilation was in Tafida’s best interests based on a number of factors.^8^ Tafida was minimally conscious and whilst there was little prospect of improvement, she was not considered to be in pain. The burden of treatment was considered to be low. There was a responsible body of medical opinion suggesting she should continue to receive treatment and that this could eventually occur at home with a loving and devoted family. Further, Justice MacDonald heard evidence that children in England in a similar position have received long term mechanical ventilation, that Tafida could be safely transferred to Italy and that there was private funding available to allow this to occur. Of note, Justice MacDonald considered the Islamic religious beliefs of the parents and of 5 year old Tafida as a relevant consideration in deciding whether it was in her best interests to die. In previous cases, the courts have rejected the relevance of parents’ religious beliefs, ‘[a]n objective balancing of [the child’s] own best interests cannot be affected by whether a parent happens to adhere to one particular belief or another, or none’.^9^ Yet despite accepting that Tafida could not have developed a sufficient understanding of Islam and of life and death to hold a view on her present position, Justice MacDonald still held that the religious tenets by which Tafida was raised should be given weight in the balancing exercise.^10^

In the months following the case, Tafida was transferred to Italy and the family has reported improvements in the form of reduced reliance on ventilation.^11^ There does not, however, appear to have been a substantial improvement in Tafida’s neurological condition and she will continue to require constant care. English and Italian clinicians agreed during the hearing that improvement was possible for Tafida and that this may allow her to go home, but the English clinicians questioned whether this was a substantial improvement.^12^ The difference was not about what was medically possible, but whether this existence was in Tafida’s interests and clearly Tafida’s parents and the Italian doctors continue to believe it is.

## 3 Potential Inconsistency with previous cases

Tafida’s parents had identified doctors willing to provide ongoing treatment to her and appeared able to fund this treatment. Although there were few benefits that could be identified in the provision of long term ventilation, it was also found that she was not experiencing any pain. As a result, Justice MacDonald understandably erred on the side of maintaining Tafida’s life and allowing the parents to raise their child in accordance with their beliefs. However, the conclusions in Tafida’s case contrast with other recent high profile cases, in which the benefits and burdens of life do not appear to have been weighed in the same way.

The case of *Evans* involved a 21 month old child, Alfie Evans, with a neurodegenerative condition that had left him in a ‘semi-vegetative state’ and unresponsive to stimuli.^13^ As Alfie’s doctors had determined ongoing mechanical ventilation was not appropriate, Alfie’s parents had found doctors in Rome willing to provide treatment. Justice Hayden held that ongoing ventilation was not in Alfie’s best interests and that he should not be transferred to Rome.^14^ Justice Hayden came to this conclusion on the basis that, since there was no prospect of improvement, ongoing ventilation was futile.^15^ Justice Hayden further identified the risks of transferring Alfie, such as a risk of infection, and noted that it was undesirable for Alfie to die in transit.^16^ Justice Hayden concluded that Alfie should not be taken to Rome because the risks were not justified given the little prospect of Alfie benefiting from treatment.^17^ However, it is unclear that the risks to Alfie of transfer were any different to those affecting Tafida Raqeeb and all experts agreed that there was also no prospect of Tafida recovering any more than minimal awareness.^18^

The case of *Haastrup* is also comparable.^19^ The case involved an 11 month old child, Isaiah, who had suffered severe hypoxic ischemic brain damage at birth. There was a consensus view from medical practitioners that there was no evidence Isaiah could interpret or interact with the outside world and so no evidence he was suffering.^20^ Doctors believed that it was in Isaiah’s best interests to withdraw mechanical ventilation and allow him to die. Isaiah’s parents disagreed with the doctor’s prognosis and conclusion about treatment. Justice MacDonald held that mechanical ventilation should be withdrawn as ‘in cases where the end of life is in issue, for many the concept of human dignity becomes encapsulated by the idea of a 'peaceful' or 'good' death’.^21^ Like Tafida, it was recognised Isaiah was not experiencing any suffering and could have been kept alive by mechanical ventilation for years. But rather than focusing on the absence of suffering, Justice MacDonald focused on the absence of benefits available to Isaiah and the likely manner of his death if treatment was continued.^22^

These cases highlight the challenges of determining the best interests of the child and the extent to which different values inform how evidence is interpreted and applied, as well as different evaluations of probabilities. The different evaluations of suffering appear to potentially reflect an inconsistency in the interpretation of evidence rather than a difference in the cases. All three children had severe neurological damage and were all unlikely to experience very much if anything at all, although in each case it was impossible to prove the absence of painful experience. In *Raqeeb*, ventilation was considered inherently beneficial because it would maintain life and life was viewed as sacred.^23^ In *Evans* and *Haastrup*, by contrast, if ventilation would not provide some additional benefit beyond merely maintaining life, this was seen as an unjustifiable assault on the child’s dignity.^24^ These differing conclusions highlight that the benefits and burdens of any medical treatment are necessarily understood through a value system; this may vary depending on which judge hears the case.

## 4 Identifying a minimally conscious child’s best interests

These three cases illustrate the difficulty of identifying the best interests of a minimally conscious child. Relevant considerations include the pleasure and suffering the child is able to derive from life, but in many of these cases this is likely to lead to an impasse. As the child is minimally conscious they are likely to experience few pleasures from life, but also little suffering, though we cannot be certain of either. Given their minimal awareness of anything, it is often difficult to identify whether the child has any interests in anything. This leads to consideration of even more difficult questions, such as what is inherently valuable in human life? Or, what is the value of a peaceful death? Or, what impact should respect for human dignity have on the provision of invasive medical treatments? Or what is the value of a small chance of a small improvement?

The challenge of assessing the best interests of an unconscious, or barely conscious, person were highlighted in *Airedale NHS Trust v Bland*.^25^ In the Court of Appeal, Lord Justice Hoffman held it was fallacious to suggest a person only has interests in things in which they may consciously experience and people have an interest in a dignified death, even though they may not experience this.^26^ This view, however, assumes a partly non-experiential account of well-being or interests, which is at least contestable. Indeed, in the House of Lords, both Lord Mustill and Lord Keith rejected Hoffman’s argument, suggesting when or how Bland died was of no consequence to him because he had no awareness of anything.^27^ Despite citing these arguments, in *Raqeeb* Justice MacDonald returned to the argument of Lord Justice Hoffman and held that it is wrong to suggest a child who experiences nothing or very little can derive no benefit from being kept alive.^28^

These questions (of the value of life, the nature of human dignity) are ones that the greatest philosophers such as Aristotle, Plato, Bentham, Nietzsche and others have struggled with for thousands of years, and unsurprisingly judges have not been able to resolve them either. As the child is barely cognisant of their existence, it is difficult to identify relevant interests of the child that favour either the provision or withdrawal of treatment. As Gillon argues, in some cases both the position of the parents and the position of the doctors may be morally justifiable.^29^ Foster has argued that given the strong presumption in favour of maintaining life in English law and the lack of certainty about the best interests of a person in a persistent vegetative state or minimally conscious state, it is always unlawful to withdraw life sustaining treatment from a person in such a state.^30^ This is because it is difficult to identify any harms in maintaining life that are strong enough to overcome the presumption. While this suggestion is extreme and provocative, it highlights the challenge of claiming that it is in an unconscious or minimally conscious person’s interests to die.

## 5 Why are the courts making these decisions?

The court generally decides these matters under its *parens patriae* jurisdiction, which gives the court jurisdiction to protect the interests of those who cannot take care of themselves.^31^ Although the court often also simultaneously invokes its jurisdiction under the *Children Act 1989*.^32^ As Lord Esher MR described the *parens patriae* jurisdiction, ‘The Court is placed in a position by reason of the prerogative of the Crown to act as supreme parent of children, and must exercise that jurisdiction in the manner in which a wise, affectionate, and careful parent would act for the welfare of the child’.^33^ Lord Donaldson MR described the inherent powers of the court exercising its *parens patriae* jurisdiction as ‘theoretically limitless’ and suggested ‘they certainly extend beyond the powers of a natural parent’, although others have described a more limited power.^34^ This power is now generally exercised through the use of declarations about what would be lawful in an individual case.

In exercising its *parens patriae* jurisdiction, the court must make a decision about what is objectively in the child’s best interests.^35^ The relevant interests extend beyond medical interests, and must include other interests such as emotional, sensory, and instinctive.^36^ The views of the child and the parents (about the child’s interests) must be considered; however, the parents’ own interests are taken to be of no relevance.^37^ There must always be a strong presumption in favour of preserving life.^38^ Beyond this, courts have taken different approaches to the most appropriate way of determining best interests. Some judges purport to be making the decision the child would make if they were able to and consider determination of the child’s best interests to be a form of substitute decision making.^39^ Others are of the view that they are required to make an objective decision about the welfare of the child and embrace the paternalistic nature of the jurisdiction.^40^ Courts have rejected attempts to define or refine the best interests approach. In *Wyatt v Portsmouth Hospital NHS Trust*, Lord Justice Wall held that in considering best interests ‘the forensic debate should, in our judgment, be unfettered by any potentially contentious glosses on the best interests test which are likely either inappropriately to shift the focus of the debate, or to restrict the broad exercise of the judicial discretion’.^41^ Lord Justice Wall cited an earlier case in which it was recognised ‘[t]he infinite variety of the human condition never ceases to surprise and it is that fact that defeats any attempt to be more precise in a definition of best interests’.^42^

Yet, it is arguable that court involvement in these cases is not strictly necessary. In *Raqeeb*, Justice MacDonald suggested it was necessary to seek a determination from the court in cases in which hospitals and parents disagreed about whether or not life sustaining treatment was appropriate for an unconscious child.^43^ However, this proposition is questionable for a number of reasons. At a fundamental level, given the remedy provided is a declaration, a court application cannot be necessary before doctors proceed. A declaration is a remedy that allows a judge to advise on the lawfulness of the proposed course of action.^44^ If a court declares withdrawal of treatment to be lawful, this withdrawal must be a lawful act. The court’s declaration does not make this the case. The judge cannot make an action that would otherwise be unlawful lawful through a declaration.^45^ So in the case of *Evans*, for example, the declaration did not make the withdrawal of treatment lawful, the declaration was an advisory statement that the doctors’ proposed course of action was lawful. The court’s involvement was not necessary for the doctors to proceed, it merely provided comfort to the parties.

Justice MacDonald suggested a declaration from the courts is necessary because if parents do not consent to the withdrawal of treatment, there would be ‘a void in relation to consent’.^46^ This mischaracterises the role of consent. Parents must consent to the provision of (non-emergency) treatment because otherwise it would be an unlawful invasion of the child’s bodily integrity.^47^ However, consent is not required for the non-provision of treatment.^48^ Whether a failure to provide treatment is lawful will be assessed under the law of negligence or criminally negligent homicide, not battery.

Regardless of the accuracy of Justice MacDonald’s statement, at a practical level hospitals are understandably concerned to act lawfully and given the consequences of a decision to withdraw life sustaining treatment appear to prefer to seek the comfort of a declaration that this would be lawful prior to proceeding. Decisions to withdraw life sustaining treatment are common in intensive care unit. A Canadian study of neonatal intensive care units found that 84% of deaths followed a decision to withdraw treatment.^49^ The difficulty arises when parents do not agree with these decisions and threaten litigation. This leave hospitals with a choice between seeking a prospective declaration their proposed course of action is lawful or proceeding and risking being sued or even criminal investigation. Unsurprisingly, hospitals often seek a prospective declaration from the court that the course of action would be in the child’s best interests.

## 6 The ignored relevant consideration

The elephant in the room in these disputes about long term mechanical ventilation of children is the question of limited resources. When these matters have come before the court, judges have concluded that their paramount concern must be the best interests of the child.^50^ Yet, this limits the extent to which the court may consider the broader context of the child’s care.

Long term mechanical ventilation is the paradigm example of highly expensive potentially life-long treatment. One analysis, published in 2006, found that long term home ventilation cost £240,000 per year, compared with £150,000 per year if patients were cared for in a paediatric ward, and £630,000 if patients received care in an intensive care unit.^51^ The standard cost limit for treatment in the UK is £30,000 per Quality Adjusted Life Year (QALY). Home ventilation vastly exceeds this. At this cost, long-term mechanical ventilation could not be cost-effective based on standard thresholds.

While the question of whether treatment was in the best interests of the children in the cases discussed is extremely difficult, the decisions by the NHS Trusts not to offer long term mechanical ventilation appears much more clear-cut when viewed in terms of resources. Long term ventilation for these children would be vastly outside the conventional cost-effectiveness threshold applied to medical treatments. At best, the benefits to the child in providing long term mechanical ventilation are marginal – the child’s life may be maintained but there was little else that could be achieved. Long term mechanical ventilation at home is very expensive, and this will inevitably mean that other costs elsewhere in the health system cannot be met. Even more crucially, intensive care beds (and carers in the community able to support home ventilation) are a *scarce* resource. Placing a child in a minimally conscious state on long term ventilation is highly likely to mean that other children (possibly with much greater potential to benefit) are unable to be admitted to intensive care, or unable to receive vital specialised nursing support at home.

In the case of Raqeeb, the family elected to move Tafida to the Italian hospital. However, the court decision in Raqeeb would have potentially obliged clinicians to continue treatment in the UK intensive care unit had the family chosen to stay. Moreover, should she return to the UK, clinicians may feel compelled to continue life-sustaining treatment.^52^ There may have been a shift over the last three decades in the court’s willingness to intervene in such cases. In *Re J* (*A minor*), Lord Donaldson MR noted ‘The court when considering what course to adopt in relation to a particular child has no knowledge of competing claims to a health authority's resources and is in no position to express any view as to how it should elect to deploy them’.^53^ In the same case Lord Justice Balcombe went even further, suggesting there were no situations in which the court should make orders that would even indirectly require doctors to treat a child contrary to their clinical judgment.^54^ The decision in Raqeeb appears to conflict with that statement.

## 7 An alternative approach

Although everyone in these cases is undoubtedly aware that treatment was being provided in a system with limited resources, the requirement to make decisions in the child’s best interests prevents explicit consideration of this. This was exemplified in *Raqeeb*, in which Justice MacDonald referred to the parents’ capacity to fund the treatment themselves eight times but did not explain how this was relevant.^55^ The case of *R v Cambridge Health Authority, Ex parte B* demonstrates how decisions could be made and what the role of the courts would be in making these decisions.^56^ The case involved a 10 year old child with cancer, for whom previous treatments had been unsuccessful and whose parents were seeking two phases of treatments that each had around a 10% chance of success and would cost £75,000. The Health Authority had determined it would not fund the treatment. As the decision was made on the basis of funding, the matter was heard as a judicial review of an administrative decision, providing more defined grounds for court involvement.

### 7.1 Decision making in hospitals

Clinical Commissioning Groups (CCGs) are responsible for the purchasing of health services in the NHS and should determine when mechanical ventilation is affordable. A CCG is required to exercise its functions ‘effectively, efficiently and economically’.^57^ And ‘health authorities of all kinds are constantly pressed to make ends meet… Difficult and agonising judgments have to be made as to how a limited budget is best allocated to the maximum advantage of the maximum number of patients’.^58^

In the NHS, CCGs determine the broad categories of health services that will be purchased and NHS Trusts provide those services in accordance with the agreed standards.^59^ In purchasing appropriate services, there is not an absolute duty to provide particular services, and the CCG is ‘entitled to have regard to the resources available to it’.^60^ At present, CCGs have not set standards or conditions on long-term ventilation. However, they could create standards about when it is appropriate to provide treatment such as mechanical ventilation. Such standards could place limits on the amount of time it is appropriate to provide mechanical ventilation or other limits based on the likely outcome of mechanical ventilation. These decisions would be made at a population level and based on the likely benefit derived from the resources expended, rather than on a case by case basis. These decisions could be facilitated by “big data” and artificial intelligence, to apply standard cost-effectiveness thresholds typically used for drugs to all medical interventions, taking account of relevant patient specific variables.^61^ This could be called “Precision Justice”, mirroring the advance of precision medicine.

If CCGs are unwilling to make these decisions at a regional level, they may also be made at a national level or through a nationally applicable process. Standards could be developed at a national level through NICE guidance, which could establish criteria for when it would be appropriate to provide long-term ventilation.^62^ If it proves too difficult to articulate criteria or guidelines that could be applicable in the range of clinical circumstances when long-term ventilation may be sought, it might be possible or preferable to establish a clear *process* for such decisions to be made. That could be through the establishment of a treatment review panel or drawing on the existing resource of ‘individual funding review panels’.^63^

It is not the aim of this paper to definitively identify how resources like long-term ventilation should be allocated. We have elsewhere outlined some elements of an ethical approach,^64^ as have others.^65^ Rather, our claim is that if and when there is a clear and defensible decision that continued mechanical ventilation is not appropriate, that this would then allow a different approach to disagreement. Changing the locus and ethical basis for decision making about treatment in the context of disputes would be valuable in a number of important ways. If there had been a clear process for identifying whether long-term ventilation for *Raqeeb*, *Evans* and *Haastrup* was appropriate – that may have changed the nature of the disagreement. Clinicians would no longer have been placed in a position of disagreeing with parents about what would be ‘best’ for the child — instead, the medical team would have been making a clear and defensible judgement that such treatment was not *appropriate* in the context of the limited resources of the healthcare system.

### 7.2 Role of the courts

If a body, such as a CCG, declined to provide treatment on the basis of resource allocation, parents may accept this decision or they may seek judicial review. As this is an administrative decision by a public body, the court would have jurisdiction but this review would proceed very differently to a case under the *parens patriae* jurisdiction. In a case of judicial review, the role of the court is not to assess how resources should be allocated (or what would be best for the child), rather it is to determine whether the decision of the CCG was lawfully made.^66^ This would be assessed on administrative law grounds. For example, a decision may be unlawful if it is so unreasonable that no reasonable CCG could have made the decision.^67^ The grounds to find the decision of the CCG unlawful would be much more limited than the court’s discretion under the *parens patriae* jurisdiction.

Any policy or decision would also need to comply with the *Human Rights Act 1998*.^68^ Again though, this would not allow the courts to simply make a decision about what they thought was best in the circumstances. CCGs have a wide discretion to make decisions about the allocation of resources, and doing so will not necessarily constitute an interference in the rights of an individual who is denied treatment because of such a policy.^69^ It is acceptable for a CCG not to fund clinically indicated treatment if there are broader policy reasons not to do so, such as the appropriate use of limited resources.^70^

## 8 Why is a distributive justice approach preferable?

### 8.1 A more ethically defensible approach

In cases involving critically ill children for whom ongoing treatment may arguably provide marginal benefit but at great expense, a distributive justice approach provides the most appropriate and equitable way of making decisions. In a diverse society, arguments about the best interests of such a child are intractable. A distributive justice approach offers a way out of this quagmire. Resource allocation decisions are made across health services every day and treatments are withheld on the basis they would not be a reasonable use of limited resources. There is no reason decisions about long term mechanical ventilation should not be made on the same basis. Decisions about the best interests of a child appear to be made in the abstract, a distributive justice approach recognises the context in which treatment decisions are made.

An advantage of a distributive justice approach is that decisions about minimally conscious children do not need to be made on notions like the intrinsic value of human life or when life is worth living. It may be recognised that every human life is intrinsically valuable, but this does not mean that it is always just and fair to maintain human life. Instead, competing demands on limited resources mean that difficult decisions need to be made. It is only reasonable that a limited medical resource should be used where it will have the greatest possible benefit. If mechanical ventilation may provide a child with an opportunity to recover and lead a full life, it seems only reasonable to prefer that child’s treatment over treatment for another child who will remain minimally conscious. It is extraordinarily difficult, if possible at all, to describe the criteria that make life not worth living. Substantial pain and suffering seem at least necessary conditions, though these were arguably absent in all three cases above. It is less controversial to compare the value of lives: a longer life is better than a shorter life. A life of less pain is better than a life of more pain. And so on. In this way, distributive justice arguments may be more tractable by comparing in relative terms the value of life, rather in than in absolute terms of whether a life is worth living.

### 8.2 A more suitable role for the courts

A distributive justice approach not only provides a way out of the quagmire created by the best interests approach in a diverse society, it also gives the courts a more suitable role through administrative law. Administrative law provides a suitable basis for assessing the appropriateness of decisions by public bodies. As the courts have identified in administrative law cases involving the provision of treatment, ‘Were we to express opinions as to the likelihood of the effectiveness of medical treatment, or as to the merits of medical judgment, then we should be far from the sphere which under our constitution is accorded to us’.^71^ This does not mean the court cannot ‘submit the decision making process to rigorous scrutiny’.^72^ But this scrutiny should be on the basis of whether or not the decision was lawful, rather than second guessing medical opinions, making contestable value judgements, or acting as a ‘wise, affectionate, and careful parent’. ^73^

The use of the *parens patriae* jurisdiction is often called for in the context of disputes relating to children because of the significance and irreversibility of the decisions being made. These are important decisions, but they must be considered in the context of the many important decisions that are made in hospitals every day. Just because a decision involves death, this should not necessitate court involvement. Even if courts make every effort to expedite such decisions, a court case will invariably delay decision making. During this time, the status quo must be maintained. The case of *Gard* is instructive. That case involved an 8 month old child with a rare mitochondrial disease who was minimally conscious and whose parents wanted to take him to the US for experimental treatment.^74^ In the six months it took for the case to be resolved through the courts, he was left on a mechanical ventilator. During this period he could have been provided with the experimental treatment his parents sought. Instead, the child spent months on mechanical ventilation waiting for the court process to be resolved. Although the court process is described as a safeguard, the practical outcome is that children are left in limbo, sometimes for months, waiting for a decision.

The other important consideration is the cost of the court process. Whilst resource arguments often focus on the costs of medical treatments, taking a matter through the courts is also a considerable expense for the state. The NHS Trust must obtain legal advice and representation, doctors must provide reports and give evidence, and holding hearings is also expensive. The cost of conducting court hearings is rarely considered in discussion of limited resources available for medical treatment, but ultimately all these costs must be covered by public funds. The administration of justice is an important public service, but this does not mean court cases are always the most effective use of public funds.

## 9 Hospital intervention in parental attempts to seek treatment elsewhere

Focusing on the appropriate use of resources may change the process according to which hospitals may intervene in parental decisions to seek treatment elsewhere. The distributive justice approach would recognise that the resources available to an NHS Trust are limited and that it may not be appropriate to expend these on long term mechanical ventilation of a child who experiences no apparent enjoyment from life. But this alternative approach may also clarify that if the parents are able to find an alternative means of accessing mechanical ventilation, there may no longer be a strong reason to prevent the child from accessing it. As George identifies, the *Children Act 1989* was enacted to govern decision making for children and provides a more appropriate framework for interfering in parental decisions focused on preventing significant _harm._^75^

On appeal in the case of *Gard*, it was argued on behalf of the parents that in order to intervene to prevent the parents from taking their child overseas, significant harm would need to be demonstrated as this is the standard in the *Children Act 1989* for intervening in parental decisions.^76^ This argument was rejected on the basis the hospital could apply under the court’s inherent jurisdiction for a declaration about treatment, at which point the court must make a determination of the child’s best interests and not a determination about whether the parent’s preferred course of action was appropriate.^77^

In the wake of the Gard case, there have been proposals for legal change to introduce a “significant harm” test for situations where parents seek treatment by other health professionals.^78^ To date, these proposals have not yet been introduced to parliament, and it is unclear whether they would be passed. However, under the proposed distributive justice approach described in this paper, such a change may be unnecessary. If a hospital had already determined that treatment was not appropriate because of resources, it is not clear it could then apply for a determination about whether treatment was in the child’s best interests. As an administrative decision would already have been made, there would be no live dispute about whether medical treatment was in the child’s best interests. If the NHS Trust wished to prevent the transfer of a child arranged by parents, an application would need to be made under the *Children Act 1989* for a care order or a supervision order to prevent ‘significant harm’ to the child in being taken overseas.^79^ It is unclear, however, whether the court would still allow an application under the *parens patriae* jurisdiction or require this higher threshold to be met.

The “significant harm” threshold for court intervention is appropriate and, in our view, is preferable to the court making a best interests judgement.^80^ As discussed above, it is not clear why the court should simply be substituting its own decisions about what it thinks is best. The revised test would not allow parents to make harmful decisions for their child, nor would it allow health professionals (whether in the UK or elsewhere) to provide or continue harmful medical treatment. However, if parents’ pursuit of medical treatment would *not* be significantly harmful, (and there are no relevant resource based concerns) it is difficult to see what possible ethical basis there could be for initiating costly and potentially distressing court proceedings.

## 10 Practical implications of a distributive justice approach

A distributive justice approach could have considerably altered the three cases discussed above. In each of the cases, the initial decision by the NHS Trust not to provide ongoing long-term mechanical ventilation may well have been appropriate if there had been a clear and fair decision not to allocate publicly funded resources for that purpose. But in some of the cases the parents were able to secure alternative funding for treatment and an alternative location for treatment. In such cases, the NHS Trust should only interfere in the parents’ decision to move their child overseas if the child would suffer significant harm as a result of the parents’ decision. Given the decisions about whether or not treatment of these children was in their best interests appeared to be so finely balanced, it may be that courts would not have concluded that the children would have been significantly harmed by the decision. That depends on how harm is conceived though and what level of harm is considered to be significant enough to warrant intervention.^81^

A shift from ‘best interests’ to ‘significant harm’ may not necessarily alter the outcome of cases. For example, in the *Gard* case Justice MacFarlane suggested this would not have made a difference, as the parents’ proposed course would cause significant harm.^82^ However, others have argued that it is hard to see how Gard’s parents were exposing him to significant harm to attempt a trial of experimental treatment provided by the world’s expert in his condition at a world leading institution.^83^ The alternative approach suggested here would mean judges were not just considering the application of different wording, but a different question. Under the *parens patriae* jurisdiction the courts are required to determine whether medical treatment is in the child’s best interests. If treatment is withheld on the basis of available resources and parents identify available treatment elsewhere, the question for the court should be whether state intervention is warranted to prevent parents causing significant harm to their child. Although it is unclear whether this different context would alter the outcome of cases, theoretically it sets a higher threshold for intervention and requires courts to justify the appropriateness of intervention.

The alternative approach suggested would also allow more economically sustainable decisions. In the case of *Raqeeb*, Justice MacDonald suggested it was appropriate to provide Tafida with mechanical ventilation because other children in England in a similar situation had been provided with treatment.^84^ To suggest that just because a treatment was made available elsewhere it should be made available by the current NHS Trust fails to account of the constraints on the particular CCG and Trust. A distributive justice approach would not apply such a high standard and would instead assess whether any reasonable CCG and Trust could have made the same decision. This avoids the difficulty of suggesting that because a treatment is provided somewhere, it must be provided everywhere. Indeed, it may be that it is unjust that mechanical ventilation is being provided in some of these other cases.

In cases such as *Evans* and *Raqeeb*, consideration would also need to be given to the long term plans for care. In *Raqeeb*, the parents had secured sufficient funding to take their daughter to Italy and for a tracheostomy that would allow home ventilation. The parents suggested they could then return to England. What was not addressed was who would support home ventilation. Given the Italian doctors suggested Tafida could be maintained on ventilation for 10-20 years, this could be very expensive, in the range of millions of pounds. Even if such children experience a normal quality of life (value 1.0) home ventilation would still fall outside the standard threshold, since it costs over £200,000 per year. The quality of life of Tafida is potentially closer to zero, rendering the intervention even less cost-effective. A distributive justice approach would allow consideration of this question and a more practical discussion about the long term care of children on home ventilation.

## 11 Limitations of a distributive justice approach

A distributive justice approach would not solve the problem that these remain extremely difficult and emotional decisions. It may be seen to simply push these decisions back to CCGs (or other resource allocation bodies) and hospitals. The distributive justice approach clearly places the burden of ethical decision making on CCGs and hospitals, but this is consistent with their role and the range of difficult decisions they are already required to make. Hospitals, perhaps assisted by clinical ethics processes, are also in a much better position to make these decisions than the courts. Hospitals have an understanding of the science, but also of their budgets and competing demands.

We have criticised the best interests approach applied by the courts under the *parens patriae* jurisdiction on the basis that attempting to identify the best interests of the child was subjective and potentially inconsistent. It may be argued that focusing on appropriate resource allocation gives rise to similar issues. The key difference is that there are existing frameworks for assessing the cost effectiveness of treatments, which are already broadly applied in health systems. These may be challenged, and there will always be borderline cases, but they are arguably more robust than judicial determinations of a child’s best interests. Moreover, such judgements are relative not absolute, as we have argued. Similar arguments may be made about the difficulty of identifying significant harm to the child. But these are also based on a well-established threshold that is applied consistently across family law.

## 12 Conclusion

Decisions about the long term ventilation or other life prolonging medical treatment of critically ill infants and children will always be difficult and there is no approach that will resolve this complexity. This complexity should not be an invitation for judicial intervention though. Instead, the courts should exercise restraint. This restraint could be encouraged by hospitals making clear and transparent decisions about why they are not offering mechanical ventilation. If a hospital plainly states that mechanical ventilation will not be provided because the resources could be more effectively utilised elsewhere, the courts may review this decision but are limited in the extent to which they may do so. Judicial review is a more appropriate role for the courts, who have no understanding of the resource limitations on hospitals and a very limited understanding of the clinical considerations for the child.

This approach would mean that in cases like *Haastrup*, *Evans* and *Raqeeb*, the hospitals may decide not to offer long term ventilation and may implement that decision without the long delay caused by court involvement. If parents opposed the decision, the extent to which the courts could intervene would be limited. But it would also mean the parents were free to pursue other options for treatment. If the health professionals wished to prevent parents from accessing treatment elsewhere, they may do so if that treatment would pose a risk of significant harm. In cases of children in minimally conscious states, whether courts would intervene to prevent treatment elsewhere would depend on how they interpreted harm and whether this was considered significant enough to warrant state intervention.^85^ This approach would recognise the value pluralism in our society and that there may be more than one view about a child’s best interests. This approach would also recognise the context in which all treatment decisions must be made and allow those best placed to understand this context to make decisions. While it is beyond the scope of this paper, this shift in approach may also be relevant to disputes about treatment for adult patients.

We should move to a distributive justice based, rather than primarily interest based, justification for limitation of life prolonging mechanical ventilation in children.

## Footnotes

1 See, for example, I. Goold, ‘Evaluating ‘Best Interests’ as a Threshold for Judicial Intervention’ in Imogen Goold, Jonathan Herring, and Cressida Auckland, *Parental Rights, Best interests and Significant Harm* (Hart Publishing: Oxford, 2019), 29

2 *National Health Services Act 2006*, s 14Q; *The Queen (on the application of SB)(by his father and litigation friend PB)* [2017] EWHC 2000 (Admin), [20]

3 J. Savulescu, ‘Is it in Charlie Gard's best interest to die?’ *Lancet* 389, 10082 (2017): 1868-1869

4 *Tafida Raqeeb v Barts NHS Foundation Trust* [2019] EWHC 2531 (Admin) and *Barts NHS Foundation Trust v Shalina Begum and Muhhamed Raqeeb and Tafida Raqeeb* [2019] EWHC 2530 (Fam)

5 *Tafida Raqeeb v Barts NHS Foundation Trust* [2019] EWHC 2531 (Admin) and *Barts NHS Foundation Trust v Shalina Begum and Muhhamed Raqeeb and Tafida Raqeeb* [2019] EWHC 2530 (Fam), [33]

6 *Tafida Raqeeb v Barts NHS Foundation Trust* [2019] EWHC 2531 (Admin) and *Barts NHS Foundation Trust v Shalina Begum and Muhhamed Raqeeb and Tafida Raqeeb* [2019] EWHC 2530 (Fam), [158]

7 *Tafida Raqeeb v Barts NHS Foundation Trust* [2019] EWHC 2531 (Admin) and *Barts NHS Foundation Trust v Shalina Begum and Muhhamed Raqeeb and Tafida Raqeeb* [2019] EWHC 2530 (Fam), [188]

8 *Tafida Raqeeb v Barts NHS Foundation Trust* [2019] EWHC 2531 (Admin) and *Barts NHS Foundation Trust v Shalina Begum and Muhhamed Raqeeb and Tafida Raqeeb* [2019] EWHC 2530 (Fam), [186]

9 *An NHS Trust v MB (A child represented by the Children and Family Court Advisory & Support Service as guardian ad litem and Mr & Mrs B (parents)* [2006] EWHC 507 (Fam), [50]

10 *Tafida Raqeeb v Barts NHS Foundation Trust* [2019] EWHC 2531 (Admin) and *Barts NHS Foundation Trust v Shalina Begum and Muhhamed Raqeeb and Tafida Raqeeb* [2019] EWHC 2530 (Fam), [184]

11 S Greenhill, ‘Tafida Raqeeb survives OFF life-support’, Daily Mail, January 9, 2020, https://www.dailymail.co.uk/news/article-7866987/Tafìda-Raqeeb-survives-life-support-Big-step-British-fìve-year-old.html

12 *Tafida Raqeeb v Barts NHS Foundation Trust* [2019] EWHC 2531 (Admin) and *Barts NHS Foundation Trust v Shalina Begum and Muhhamed Raqeeb and Tafida Raqeeb* [2019] EWHC 2530 (Fam), [171]

13 *Alder Hey Children’s NHS Foundation Trust v Thomas Evans, Kate James, and Alfie Evans (A child by his guardian)* [2018] EWHC 308 (Fam)

14 *Alder Hey Children’s NHS Foundation Trust v Thomas Evans, Kate James, and Alfie Evans (A child by his guardian)* [2018] EWHC 308 (Fam), [66]

15 *Alder Hey Children’s NHS Foundation Trust v Thomas Evans, Kate James, and Alfie Evans (A child by his guardian)* [2018] EWHC 308 (Fam), [51]

16 *Alder Hey Children’s NHS Foundation Trust v Thomas Evans, Kate James, and Alfie Evans (A child by his guardian)* [2018] EWHC 308 (Fam), [63]

17 *Alder Hey Children’s NHS Foundation Trust v Thomas Evans, Kate James, and Alfie Evans (A child by his guardian)* [2018] EWHC 308 (Fam), [64]

18 *Tafida Raqeeb v Barts NHS Foundation Trust* [2019] EWHC 2531 (Admin) and *Barts NHS Foundation Trust v Shalina Begum and Muhhamed Raqeeb and Tafida Raqeeb* [2019] EWHC 2530 (Fam), [31]-[34]

19 *Kings College Hospital NHS Foundation Trust v Teksha Thomas and Lanre Haastrup and Isaiah Haastrup* [2018] EWHC 127 (Fam)

20 *Kings College Hospital NHS Foundation Trust v Teksha Thomas and Lanre Haastrup and Isaiah Haastrup* [2018] EWHC 127 (Fam), [36]

21 *Kings College Hospital NHS Foundation Trust v Teksha Thomas and Lanre Haastrup and Isaiah Haastrup* [2018] EWHC 127 (Fam), [109]

22 *Kings College Hospital NHS Foundation Trust v Teksha Thomas and Lanre Haastrup and Isaiah Haastrup* [2018] EWHC 127 (Fam), [107]-[108]

23 *Tafida Raqeeb v Barts NHS Foundation Trust* [2019] EWHC 2531 (Admin) and *Barts NHS Foundation Trust v Shalina Begum and Muhhamed Raqeeb and Tafida Raqeeb* [2019] EWHC 2530 (Fam), [182]

24 *Alder Hey Children’s NHS Foundation Trust v Thomas Evans, Kate James, and Alfie Evans (A child by his guardian)* [2018] EWHC 308 (Fam), [64]; *Kings College Hospital NHS Foundation Trust v Teksha Thomas and Lanre Haastrup and Isaiah Haastrup* [2018] EWHC 127 (Fam), [107]-[109]

25 *Airdale NHS Trust v Bland* [1993] AC 789

26 *Airdale NHS Trust v Bland* [1993] AC 789, 829

27 *Airdale NHS Trust v Bland* [1993] AC 789, 858 and 897

28 *Tafida Raqeeb v Barts NHS Foundation Trust* [2019] EWHC 2531 (Admin) and *Barts NHS Foundation Trust v Shalina Begum and Muhhamed Raqeeb and Tafida Raqeeb* [2019] EWHC 2530 (Fam), [172]

29 R. Gillon, ‘Why Charlie Gard’s parents should have been the decision-makers about their son’s best interests’ *Journal of Medical Ethics* 44 (2018): 462-465

30 C. Foster, ‘It is never lawful or ethical to withdraw life-sustaining treatment from patients with prolonged disorders of consciousness’ *Journal of Medical Ethics* 45 (2019): 265-270

31 R. George, ‘The Legal Basis of the Court s Jurisdiction to Authorise Medical Treatment of Children’ in Imogen Goold, Jonathan Herring, and Cressida Auckland, *Parental Rights, Best Interests and Significant Harm* (Hart Publishing: Oxford, 2019), 69

32 R. George, ‘The Legal Basis of the Court s Jurisdiction to Authorise Medical Treatment of Children’ in Imogen Goold, Jonathan Herring, and Cressida Auckland, *Parental Rights, Best Interests and Significant Harm* (Hart Publishing: Oxford, 2019), 69

33 *R v Gyngall* [1893] 2 QB 232, 241-242

34 *Re W (Minor) (Medical Treatment)* [1992] 4 All ER 627, [50]; *Kings College Hospital NHS Foundation Trust v Y (by her children’s guardian), MH* [2015] EWHC 1920, [34]

35 *Re A (A Child)* [2016] EWCA 759, [39]

36 *An NHS Trust v MB (A child represented by the Children and Family Court Advisory & Support Service as guardian ad litem and Mr & Mrs B (parents)* [2006] EWHC 507 (Fam), [16]

37 *An NHS Trust v MB (A child represented by the Children and Family Court Advisory & Support Service as guardian ad litem and Mr & Mrs B (parents)* [2006] EWHC 507 (Fam), [50]; *Re A (A Child)* [2016] EWCA 759

38 *Kings College Hospital NHS Foundation Trust v Teksha Thomas and Lanre Haastrup and Isaiah Haastrup* [2018] EWHC 127 (Fam), [69]; *Re A (Children) (Conjoined Twins: Medical Treatment)* [2000] HRLR 721, 760

39 *A Local Health Board v Y (By his children’s guardian) & Ors* [2016] EWHC 206, [19]

40 *Great Ormond Street Hospital v Constance Gard, Chris Gard & Charles Gard (A child, by his guardian ad litem)* [2017] EWHC 972 (Fam), [36]

41 *Wyatt v Portsmouth Hospital NHS Trust* [2005] EWCA Civ 1181, [86]

42 *Wyatt v Portsmouth Hospital NHS Trust* [2005] EWCA Civ 1181, [53]

43 *Tafida Raqeeb v Barts NHS Foundation Trust* [2019] EWHC 2531 (Admin) and *Barts NHS Foundation Trust v Shalina Begum and Muhhamed Raqeeb and Tafida Raqeeb* [2019] EWHC 2530 (Fam), [109]

44 *R (Burke) v General Medical Council* [2005] EWCA Civ 1003, [80]

45 *R (Burke) v General Medical Council* [2005] EWCA Civ 1003, [71]

46 *Tafida Raqeeb v Barts NHS Foundation Trust* [2019] EWHC 2531 (Admin) and *Barts NHS Foundation Trust v Shalina Begum and Muhhamed Raqeeb and Tafida Raqeeb* [2019] EWHC 2530 (Fam), [109]

47 *Re J (A Minor) (Wardship: Medical Treatment)* [1991] Fam 33, 41

48 *R (Burke) v General Medical Council* [2005] EWCA Civ 1003, [50]. While the case of *Glass v United Kingdom* is often referred to as establishing a requirement to obtain agreement, the European Court concluded the failure to obtain consent to provide diamorphine constituted a breach of the Convention. Having reached this conclusion, the Court did not consider it necessary to separately consider the issue of the hospital unilaterally placing a DNR on the patient’s file*. Glass v United Kingdom* (App No 61827/00) [2004] 77 BMLR 120, [82] and [83]

49 J. Hellman, R. Knighton, S. K. Lee, P. S. Shah, ‘Neonatal deaths: prospective exploration of the causes and process of end-of-life decisions’, *Archives of Disease in Childhood Fetal & Neonatal Ed* 101 (2016): F1-F6

50 *Wyatt v Portsmouth Hospital NHS Trust* [2005] EWCA Civ 1181, [87]

51 J. Noyes, C. Godfrey, and J Beecham, ‘Resource use and service costs for ventilator-dependent children and young people in the UK’ *Health & Social Care in the Community* 14, 6 (2006): 508-522

52 Recent news reports indicate that Tafida is no longer in an intensive care unit, but has been transferred to a rehabilitation ward (still mechanically ventilated). ‘Tafida Raqeeb: Brain-damaged girl in High Court case out of intensive care’ *BBC News*, January 10, 2020, https://www.bbc.co.uk/news/uk-england-london-51055153. This reduces, but does not eliminate, the resource concern, since her long-term ventilation in a ward, or at home, would still potentially carry a substantial cost relative to the benefit accrued.

53 *Re J (A minor) (Child in care: medical treatment)* [1993] Fam 15, 28

54 *Re J (A minor) (Child in care: medical treatment)* [1993] Fam 15, 29

55 *Tafida Raqeeb v Barts NHS Foundation Trust* [2019] EWHC 2531 (Admin) and *Barts NHS Foundation Trust v Shalina Begum and Muhhamed Raqeeb and Tafida Raqeeb* [2019] EWHC 2530 (Fam), [13], [16], [75], [78], [79], [179], [182], [186]

56 *R v Cambridge Health Authority, Ex parte B* [1995] 1 WLR 898

57 *National Health Services Act 2006*, s 14Q; *The Queen (on the application of SB)(by his father and litigation friend PB)* [2017] EWHC 2000 (Admin), [20]

58 *R v Cambridge Health Authority, Ex parte B* [1995] 1 WLR 898, 906; *R (JF, by her litigation friend RW) v NHS Sheffield Clinical Commissioning Group* [2014] EWHC 1345 (Admin)

59 D. Lock and H. Gibbs, *NHS Law and Practice*, (LAG Education and Services: London, 2018), 6-9

60 *R JF, by her litigation friend RW) v NHS Sheffield Clinical Commissioning Group* [2014] EWHC 1345 (Admin), [43]

61 D. Wilkinson, S. Petrou, and J. Savulescu, ‘Rationing potentially inappropriate treatment in newborn intensive care in developed countries’ *Seminars in Fetal and Neonatal Medicine* 23, 1 (2018): 52-58; D.Wilkinson, S. Petrou, and J. Savulescu, ‘Expensive Care? Resource-based thresholds for potentially inappropriate treatment in intensive care’ *Monash Bioethics Review* 35 (2018) 2-23

62 See for factors relevant to making such a framework S Ray, J Brierley, A. Bush, J. Fraser, G. Halley, E. Harrop, L. Casanueva, ‘Towards developing an ethical framework for decision making in long-term ventilation in children’ *Archives of Disease in Childhood* 103 (2018)

63 D. Wilkinson and J. Savulescu, *Ethics, Conflict and Medical Treatment for Children; From Disagreement to Dissensus*, (Elsevier: Edinburgh, 2019), 126-129

64 D. Wilkinson, S. Petrou, and J. Savulescu, ‘Rationing potentially inappropriate treatment in newborn intensive care in developed countries’ *Seminars in Fetal and Neonatal Medicine* 23, 1 (2018): 52-58; D.Wilkinson, S. Petrou, and J. Savulescu, ‘Expensive Care? Resource-based thresholds for potentially inappropriate treatment in intensive care’ *Monash Bioethics Review* 35 (2018) 2-23

65
N. Daniels, *Just Health: meeting health needs fairly*, (Cambridge University Press: Cambridge, 2008)

66 *R v Cambridge Health Authority, Ex parte B* [1995] 1 WLR 898, 905

67 *R v Ministry of Defence ex p Smith* [1996] QB 517, 554E; *The Queen on the application of Ann Marie Rogers v Swindon NHS Primary Care ‘Trust* [2006] EWCA Civ 392, [55]-[60]

68 *Human Rights Act 1998*, (UK), s 6

69 *R (Condliff) v North Staffordshire Primary Care Trust* [2011] EWCA Civ 910, [30]

70 David Lock and Hannah Gibbs, *NHS Law and Practice*, (LAG Education and Services: London, 2018), 11

71 *R v Cambridge Health Authority, Ex parte B* [1995] 1 WLR 898, 905

72 *The Queen on the application of Colin Ross v West Sussex Primary Care Trust* [2008] EWHC 2252 (Admin), [36]

73 R. George, ‘The Legal Basis of the Courf s Jurisdiction to Authorise Medical Treatment of Children’ in Imogen Goold, Jonathan Herring, and Cressida Auckland, *Parental Rights, Best Interests and Significant Harm*, Hart Publishing, Oxford, (2019)

74 *Great Ormond Street Hospital v Constance Gard, Chris Gard & Charles Gard (A child, by his guardian ad litem)* [2017] EWHC 972 (Fam)

75 R. George, ‘The Legal Basis of the Court’s Jurisdiction to Authorise Medical Treatment of Children’ in Imogen Goold, Jonathan Herring, and Cressida Auckland, *Parental Rights, Best Interests and Significant Harm* (Hart Publishing: Oxford, 2019)

76 *Constance Yates & Christopher Gard v Great Ormond Street Hospital for Children NHS Foundation Trust* [2017] EWCA Civ 410, [56]-[71]

77 *Constance Yates & Christopher Gard v Great Ormond Street Hospitalfor Children NHS Foundation Trust* [2017] EWCA Civ 410, [89]-[109]

78 See, for example, https://www.thecharliegardfoundation.org/charlies-law/

79 *Children Act 1989*, s 31

80 D. Wilkinson, ‘In Defence of a Conditional Harm Threshold Test for Paediatric Decision-Making’ in Imogen Goold, Jonathan Herring, and Cressida Auckland, *Parental Rights, Best Interests and Significant Harm* (Hart Publishing: Oxford, 2019)

81 D. Wilkinson, ‘In Defence of a Conditional Harm Threshold Test for Paediatric Decision-Making’ in Imogen Goold, Jonathan Herring, and Cressida Auckland, *Parental Rights, Best Interests and Significant Harm* (Hart Publishing: Oxford, 2019), 102 - 105

82 D. Wilkinson, ‘In Defence of a Conditional Harm Threshold Test for Paediatric Decision-Making’ in Imogen Goold, Jonathan Herring, and Cressida Auckland, *Parental Rights, Best Interests and Significant Harm* (Hart Publishing: Oxford, 2019), 103

83 J. Savulescu and P. Singer, ‘Unpicking what we mean by best interests in light of Charlie Gard’ 2 August 2017, *BMJ Opinion*, https://blogs.bmj.com/bmj/2017/08/02/unpicking-what-we-mean-by-best-interests-in-light-of-charlie-gard/

84 *Tafida Raqeeb v Barts NHS Foundation Trust* [2019] EWHC 2531 (Admin) and *Barts NHS Foundation Trust v Shalina Begum and Muhhamed Raqeeb and Tafida Raqeeb* [2019] EWHC 2530 (Fam), [168]

85 D. Wilkinson, ‘In Defence of a Conditional Harm Threshold Test for Paediatric Decision-Making’ in Imogen Goold, Jonathan Herring, and Cressida Auckland, *Parental Rights, Best Interests and Significant Harm* (Hart Publishing: Oxford, 2019), 102 - 105
